# From a female perspective: plyometric training’s impact on jump, sprint, and change-of-direction performance in adult female athletes—a systematic review and meta-analysis

**DOI:** 10.3389/fphys.2025.1633089

**Published:** 2025-09-15

**Authors:** Rongting Zhao, Jiwei Yao, Yangjian Dong

**Affiliations:** ^1^ College of Physical Education and Health, Guangxi Normal University, Guilin, China; ^2^ College of Physical Education, China Three Gorges University, Yichang, China

**Keywords:** plyometric training, female, jump performance, sprint performance, change-of-direction performance

## Abstract

Research on female subjects in sports science remains insufficient, particularly regarding how plyometric training affects adult female athletes’ jumping, sprinting, and change-of-direction (COD) performance. This gap has prevented definitive conclusions about the magnitude and characteristics of such performance effects. This study systematically investigates the impact of plyometric training on adult female athletes' jumping, sprinting, and COD performance. A comprehensive literature search was conducted across PubMed, Web of Science (including all databases), MEDLINE, Cochrane Central Register of Controlled Trials (CENTRAL), Embase, and SPORTDiscus, with the search time frame extending from the inception of each database to 10 May 2025. Data analysis was performed using Stata 15 software, and the methodological quality of the included studies was assessed using the PEDro scale. The results indicate that plyometric training significantly enhances the jumping performance (SMD = 0.70, p < 0.001, medium effect), sprinting performance (SMD = −0.61, p < 0.001, medium effect), and COD performance (SMD = −0.86, p < 0.001, large effect) of adult female athletes. Subgroup analysis further reveals that plyometric training significantly improves countermovement jump (CMJ; SMD = 0.84, p < 0.001, large effect), squat jump (SJ; SMD = 0.41, p = 0.046, small effect), and standing long jump (SLJ; SMD = 0.45, p = 0.031, small effect) performance, as well as sprinting performance over distances of 10 m (SMD = −0.55, p = 0.016, medium effect), 20 m (SMD = −0.55, p = 0.002, medium effect), and 30 m (SMD = −0.72, p = 0.002, medium effect). This study demonstrates that plyometric training effectively improves the jumping, sprinting, and COD performance of adult female athletes. It is recommended that coaches and athletes incorporate plyometric training into their specialized training programs to optimize sport performance and training outcomes in female athletes.

## Introduction

The significance of jumping, sprinting, and change-of-direction (COD) performance for athletes cannot be overstated, as these abilities are fundamental to numerous sports and often serve as key determinants of athletic competitiveness (Lloyd et al., 2016). For basketball players, frequent direction changes and jumps are necessary for rapid transitions between offense and defense (Svilar et al., 2018). Volleyball players need to jump frequently to spike and block (Powers, 1996). Soccer players usually have to sprint repeatedly to break away from opponents and create scoring chances (von Lieres Und Wilkau et al., 2020). Beyond their role in specific sport performance, these abilities also significantly impact athletes’ overall physical fitness and match adaptability (Haugen et al., 2019). These movements are not only practical indicators of athletic prowess, but also rooted in neuromuscular adaptations, such as motor unit recruitment efficiency and intermuscular coordination, which are critical for explosive force production (Cannon and Cafarelli, 1987). Therefore, a training method that can effectively enhance jumping, sprinting, and COD performance is essential.

Plyometric training is a scientifically proven, highly effective method to enhance jumping, sprinting, and COD performance, with wide application across sports (Ramirez-Campillo et al., 2020a; Chen et al., 2024; Chen et al., 2023a; Zhang et al., 2025; Asadi, 2013; Váczi et al., 2013; Chaabene et al., 2021). Numerous systematic reviews and meta-analyses have also reached the same conclusion. For example, Sáez-Sáez de Villarreal et al. (2010) confirmed the effectiveness of plyometric training on strength performance, Markovic (2007) verified its effectiveness on vertical jump height, and Asadi et al. (2016) demonstrated its effectiveness on COD performance. The high efficacy of plyometric training is attributed to a unique physiological mechanism known as the stretch-shortening cycle (SSC), which comprises three phases: eccentric, isometric, and concentric. This brief isometric phase acts as a transitional period to maintain muscle tension before the concentric phase (Turner and Jeffreys, 2010). During the eccentric phase, muscles lengthen under load and store elastic potential energy, rapidly released during the subsequent concentric phase when the transition between eccentric and concentric phases is rapid (Asmussen and Bonde-Petersen, 1974; Cavagna et al., 1965). During the eccentric phase, muscles utilize the stretch reflex mechanism to augment the force produced during the concentric contraction, where rapid lengthening of muscles activates sensory receptors to trigger a more forceful concentric contraction (Bosco et al., 1982; Bosco and Komi, 1979; Chen et al., 2023b). This mechanism aligns with fundamental biomechanical principles of force transfer and impulse generation, optimizing movement efficiency by synchronizing elastic energy utilization and neuromuscular activation (Kallerud and Gleeson, 2013). Therefore, plyometric training is a broadly effective physical training method that can optimize sport performance.

Although the efficacy of plyometric training is widely recognized, the representation of female athletes in sports science research remains severely limited (Paul et al., 2023). A review by Paul et al. (2023) demonstrated that from 2017 to 2021, among the top six sports medicine journals, 70.7% of studies focused on male subjects, while only 8.8% were centered on female subjects. This underrepresentation is also evident in research on plyometric training, where the effects of such training on female subjects remain inconclusive (Chen et al., 2025a). Chen et al. (2025a), in their study on plyometric training, similarly emphasized the need for increased attention to female subjects due to their scarcity in research. Female-specific factors like hormonal variations (e.g., estrogen fluctuations) may modulate neuromuscular responses to plyometric stimuli, altering motor unit firing patterns and muscle activation thresholds (Sieck and Mantilla, 2004). Such sex-related differences in training adaptations are not unique to plyometrics; as (Roberts et al. (2020) noted in their systematic review, resistance training induces similar effects on muscle hypertrophy and lower limb strength gains in males and females. Yet, females exhibit greater improvements in upper limb strength compared to males. This further supports that physiological disparities can lead to divergent training outcomes between sexes. Biomechanically, differences in lower limb kinematics and joint stiffness between sexes could also affect SSC dynamics, influencing performance gains and injury risk profiles (Hewett et al., 2005).

Given the critical importance of jumping, sprinting, and COD performance for athletes; the established efficacy of plyometric training in enhancing these abilities; and the uncertainty surrounding the effects of plyometric training on female subjects, this study aims to systematically investigate the impact of plyometric training on the jumping, sprinting, and COD performance of adult female athletes. It is hoped that the findings will provide robust support for the plyometric training practices of female athletes.

## Methods

### Eligibility criteria

This systematic review and meta-analysis was conducted by the PICOS (Participants, Interventions, Comparisons, Outcomes, and Study design) principles to establish the criteria for the inclusion and exclusion of studies. The specific inclusion and exclusion criteria are detailed in Table 1.

**TABLE 1:** Criteria for studies inclusion and exclusion.

Category	Inclusion criteria	Exclusion criteria
Population	All subjects were healthy adult female athletes, with their “healthy” status determined by reports in the included studies (as direct access to participants' information was unavailable). Their average age exceeded 18 years, meeting the World Health Organization’s criteria for adulthood (World Health Organization, 2023)	Non-female subjects, non-athlete populations, studies that did not specify the sport, subjects outside the adult age range, and unhealthy populations
Intervention	Plyometric training methodologies encompass various forms of exercise, such as unilateral and bilateral jumping movements, continuous bounding and hopping, as well as a range of drills that leverage pre-stretching, countermovement, and SSC principles (Moran et al., 2021; Chu and Myer, 2013; Ramirez-Campillo et al., 2020b)	Subjects received not only plyometric training but also other interventions such as resistance training or high-intensity interval training
Comparator	All subjects in the included studies were athletes. The control group only underwent sport-specific training	The control group underwent not only sport-specific training but also other training interventions
Outcome	The outcome measures should include assessment methods for jump height, sprint time, and COD time	Studies lacking baseline or post-test data, and for which the data could not be obtained from the corresponding author before publication
Study design	Randomized and non-randomized trials would be considered	Case report, animal studies, reviews. protocols, patents, theses, conference abstracts

SSC, stretch-shortening cycle; COD, change-of-direction.

### Search strategy

This systematic review and meta-analysis comprehensively searched six authoritative databases: PubMed, Web of Science (all databases), MEDLINE, CENTRAL, Embase, and SPORTDiscus. The search was limited to the period from the inception of each database to 10 May 2025. The search topics focused on plyometric training, females, athletes, jumping, sprinting, and change-of-direction, with Boolean logic operators (“OR” and “AND”) used to connect search terms across different themes. The study has been registered in the International Prospective Register of Systematic Reviews (PROSPERO: CRD420251049911), and the registration protocol can be accessed via the following link: https://www.crd.york.ac.uk/PROSPERO/view/CRD420251049911. The specific search strategy is detailed in Table 2, and the complete search details for all databases are provided in Supplementary Material S1 for reference.

**TABLE 2:** Search strategy and details.

Databases	Search strategy
PubMed, Web of Science (all database), MEDLINE, CENTRAL, Embase, SPORTDiscus	#1 AB = [Plyometric Exercise (MeSH Terms)] #2 AB = (“Female” OR “Woman”) #3 AB = (“player” OR “athlete”) #4 AB = (“Lower limb explosive strength” OR “Explosive strength” OR “explosive force” OR “Explosive power” OR “power” OR “Countermovement jump” OR “CMJ” OR “squat jump” OR “SJ” OR “standing long jump” OR “SLJ” OR “drop jump” OR “DJ” OR “sprint performance” OR “10 m” OR “20 m” OR “30 m” OR “50 m” OR “vertical jump” OR “VJ” OR “change of direction” OR “cod” OR “t-test” OR “t-drill” OR “505 test” OR “Illinois agility test” OR “5–10–5 test” OR “pro agility shuttle” OR “20-yard shuttle” OR “5–10–5 shuttle”) #5 #1 AND #2 AND #3 AND #4

CMJ, countermovement jump; SJ, squat jump; SLJ, standing long jump; VJ, vertical jump; 10 m, 10 m sprint; 20 m, 20 m sprint; 30 m, 30 m sprint; 50 m, 50 m sprint.

### Data extraction

The data extraction process involved three co-authors and focused on two main themes: data extraction and extraction of study characteristics. Data extraction was conducted by two co-authors (R.Z and J.Y.), who collected the means and standard deviations of both baseline and post-intervention data. Extraction of study characteristics was also performed by two co-authors (R.Z. and J.Y.), who gathered information on the first author, publication year, sample size, participant age, sport specialization, intervention duration, intervention frequency, and duration of each training session. Data extraction forms are provided in Supplementary Material S3 for reference. After completing the data and study characteristic extractions, the two co-authors (R.Z. and J.Y.) cross-checked their extracted information. In the event of discrepancies, a third co-author (Y.D.) made the final decision. The GetData software was utilized to extract unconventional data, such as that presented in error bars or bar charts. If baseline and post-intervention data could not be directly obtained or indirectly extracted using GetData, one co-author (R.Z.) contacted the corresponding authors of the included studies via email or the ResearchGate platform to request the data. If the data could not be obtained prior to publication, the study was excluded. Outcome metrics are detailed in Table 3.

**TABLE 3:** Details of outcome measures.

Outcome categories	Test
Jump performance	Countermovement jump (CMJ)
	Squat jump (SJ)
	Standing long jump (SLJ)
Sprinting performance	10 m sprint
	20 m sprint
	30 m sprint
Change-of-direction performance	Illinois agility test
	T-test
	505 test

### Methodological quality of the included studies

The methodological quality of all studies included in this systematic review and meta-analysis was assessed using the PEDro scale. The PEDro scale ranges from 0 to 10, with higher scores indicating better methodological quality. The methodological quality assessment was independently conducted by two co-authors (R.Z. and J.Y.), and the results were verified and finalized by a third co-author (Y.D.). The methodological quality ratings on the PEDro scale are interpreted as follows: poor (<4), fair (4–5), good (6–8), and excellent (9–10).

### Risk of bias assessment

The Rob 2.0 framework (Revised Cochrane Risk of Bias Tool for Randomized Trials) was employed to evaluate the risk of bias in this study. This tool assesses multiple key domains, including the generation of random sequences, the concealment of allocations, blinding procedures, handling of missing outcome data, and selective reporting of outcomes (Sterne et al., 2019).

Based on the methodological reliability, the studies were classified into three distinct levels:

1. Low risk of bias: All assessed domains were determined to be at low risk.
2. High risk of bias: The presence of any domain rated as high risk resulted in this classification.
3. Some concerns: This category applied to studies that did not meet the low or high risk of bias criteria.

Two co-authors (R.Z. and J.Y.) independently carried out the assessment process. In cases where discrepancies arose, a third co-author (Y.D.) was consulted to provide final arbitration and resolve the disagreements.

### Synthesis methods

Data analysis for this systematic review and meta-analysis was conducted using Stata (Release 15; StataCorp, United States). To avoid bias resulting from the omission of baseline scores in the pooled analysis, both baseline and post-intervention scores were included in the forest plots to ensure accurate reporting of results. Given the variability in testing equipment and protocols, the standardized mean difference (SMD) with a 95% confidence interval was used to calculate the pooled effect sizes, with p < 0.05 indicating statistical significance. The effect size of SMD can be interpreted as follows: trivial (SMD < 0.20), small (0.20 ≤ SMD < 0.50), medium (0.50 ≤ SMD < 0.80), and large (SMD ≥ 0.80) (Hedges and Olkin, 2014). Since all participants in the included studies were athletes, the potential confounding effects of sport-specific training were considered. Therefore, this study included the baseline and post-intervention means and standard deviations for the experimental and control groups. To better assess the effects of the intervention between the two groups, we artificially converted the data into change scores and standard deviations. The calculation formula is as follows: ${\text{SD}}_{E,\text{change}}=\sqrt{{\text{SD}}_{E,\text{baseline}}^{2}+{\text{SD}}_{E,\text{final}}^{2}‐\left(2\times \text{Corr}\times {\text{SD}}_{E,\text{baseline}}\times {\text{SD}}_{E,\text{final}}\right)}$ (Higgins and Green, 2008). Subgroup analyses were performed to present the summarized results for different outcomes, as detailed in Table 3. Subgroup analyses were only conducted when the number of studies within a subgroup exceeded four, and subgroup data were presented accordingly (Chen et al., 2025a). For example, subgroups for jump and sprint performance were included, and their data were presented because each subgroup contained more than four studies. In contrast, the subgroup for change-of-direction performance had fewer than four studies; therefore, it was included in the analysis but without presenting subgroup data. Although the Cochrane Handbook provides no specific guidance on selecting statistical models, we chose a random-effects model due to the diversity of subjects, variability in sports science intervention protocols, and fixed-effects models' potential to fail to account for heterogeneity (Higgins and Green, 2008). The I^2^ statistic was used to assess heterogeneity in the meta-analysis, with the following interpretation: negligible heterogeneity (I^2^ < 25%), moderate heterogeneity (I^2^ = 25%–75%), and high heterogeneity (I^2^ > 75%), with p < 0.05 indicating significant heterogeneity (Higgins et al., 2003). When high heterogeneity was detected, the stepwise exclusion method was employed to identify the source of heterogeneity. Funnel plots were used for an initial assessment of publication bias among the included studies, and Egger’s test was conducted for a quantitative evaluation of publication bias. If Egger’s test indicated significant publication bias, the trim-and-fill method was used to supplement missing studies. Additionally, to assess the impact of outliers and risk of bias on the stability of the results, we employed sensitivity analysis to evaluate the influence of the included studies on the stability of the results (Higgins and Green, 2008).

## Results

### Study selection

**FIGURE 1:**
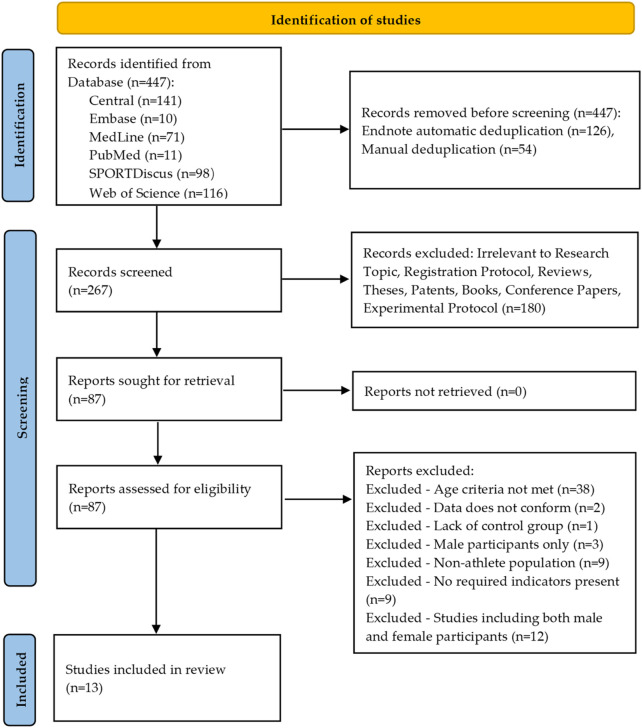
Visualizes the study selection process.

The study selection process was strictly conducted according to the inclusion and exclusion criteria, with the specific workflow presented in Figure 1. The initial search yielded a total of 447 studies. EndNote software was used for automatic and manual exclusion of duplicates, with 126 studies automatically removed and an additional 54 studies manually excluded. After removing duplicates, the remaining studies were screened based on their titles and abstracts. One hundred eighty studies were excluded at this stage, with the reasons for exclusion detailed in Figure 1. Of these, 74 studies were excluded due to the following reasons: age criteria not met, data not conforming (e.g., the study by Paktas (2021) only reported means without accompanying standard deviations), lack of a control group, male participants only, non-athlete population, no required indicators present (e.g., the study by Alikhani (Alikhani et al., 2019) did not include the tests outlined in Table 3), and studies including both male and female participants. Ultimately, 13 studies were included in the analysis. The reasons for inclusion and exclusion for all downloaded studies are documented and provided in Supplementary Material S2 for reference.

### Description of the included studies

This systematic review and meta-analysis strictly adhered to the inclusion and exclusion criteria based on the PICOS principles, ultimately including 13 studies for statistical analysis (Table 4). All participants in the included studies were adult female athletes, with the lowest mean age in the experimental groups being 18.3 ± 2.6 years and the highest being 26.5 ± 6.9 years. In all studies, the experimental groups underwent plyometric training in addition to their specific sport training, while the control groups participated in the same sport-specific training without plyometric training.

**TABLE 4:** Characteristics of included studies.

Study	Sample size (age)		Experimental group			Control group	Competitive level	Plyometric types
	PT	CON	Interventions	PT duration	Weeks × weekly sessions	Interventions		
Campo et al. (2009)	10 (22.8 ± 2.1)	10 (23.0 ± 3.2)	PT + Soccer	40–60 min	12 × 3	Soccer	Spanish National Women’s First Division	Vertical, Horizontal, Bilateral
Sánchez-Sixto et al. (2021)	11 (22.6 ± 3.2)	12 (22.6 ± 7.3)	PT + Basketball	35 min	6 × 2	Basketball	Minimum basketball training experience of 5 years	Vertical, Horizontal, Bilateral
Rosas et al. (2017)	8 (22.8 ± 2.1)	9 (24.0 ± 2.7)	PT + Soccer	—	6 × 2	Soccer	Amateur female soccer players	Vertical, Horizontal, Bilateral
Ramírez-Campillo et al. (2016)	19 (22.4 ± 2.4)	19 (20.5 ± 2.5)	PT + Soccer	40 min	6 × 2	Soccer	Amateur female soccer teams competing at the national level	Vertical, Horizontal, Unilateral, Bilateral
(PJT-1) Ramirez-Campillo et al. (2018)	8 (22.8 ± 4.3)	7 (20.1 ± 1.8)	PT + Soccer	6–20 min	8 × 1	Soccer	Amateur female soccer players	Vertical, Horizontal, Unilateral, Bilateral
(PJT-2) Ramirez-Campillo et al. (2018)	8 (21.4 ± 2.5)	7 (20.1 ± 1.8)	PT + Soccer	6–20 min	8 × 2	Soccer	Amateur female soccer players	Vertical, Horizontal, Unilateral, Bilateral
Ozbar et al. (2014)	9 (18.3 ± 2.6)	9 (18.0 ± 2.0)	PT + Soccer	30–40 min	8 × 1	Soccer	University Sports Club female soccer team that plays at Women Second League	Vertical, Horizontal, Bilateral
Ozbar (2015)	10 (19.4 ± 1.6)	10 (19.1 ± 1.7)	PT + Soccer	40 min	10 × 2	Soccer	University Sports Club female soccer team plays at Women 1st League	Vertical, Horizontal, Bilateral
Nonnato et al. (2022)	8 (23.0 ± 4.0)	8 (23.0 ± 4.0)	PT + Soccer	20–25 min	12 × 1	Soccer	Professional female soccer players w	Vertical, Horizontal, Bilateral
Maciejczyk et al. (2021)	8 (21.0 ± 3.0)	9 (18.2 ± 1.8)	PT + Soccer	—	4 × 2	Soccer	Poland 1st league	Vertical, Horizontal, Unilateral, Bilateral
Kale (2016)	10 (20.4 ± 3.0)	9 (19.4 ± 3.3)	PT + Handball	—	6 × 2	Handball	Super League Team	Vertical, Horizontal, Bilateral
Gjinovci et al. (2017)	21 (21.8 ± 2.1)	20 (21.8 ± 2.1)	PT + Volleyball	25–40 min	12 × 2	Volleyball	Highest competitive level	Vertical, Horizontal, Unilateral, Bilateral
Fischetti et al. (2019)	14 (26.5 ± 6.9)	14 (26.7 ± 5.3)	PT + Soccer	45–65 min	12 × 3	Soccer	Italian Women’s series A League	Vertical, Horizontal, Bilateral
Cherni et al. (2021)	15 (20.9 ± 2.4)	12 (21.0 ± 3)	PT + Basketball	—	8 × 2	Basketball	Elite female basketball players competing in the First Division National League	Vertical, Horizontal, Bilateral

PT, plyometric training, min, minute; PJT-1, one session plyometric jump training per-week; PJT-2, two session plyometric jump training per-week.

### Methodological quality of the included studies

The methodological quality of all included studies was assessed using the PEDro scale (Table 5). Three studies scored five points (Ozbar, 2015; Kale, 2016; Gjinovci et al., 2017), indicating fair methodological quality. The remaining nine studies scored over six points, indicating good methodological quality. The overall average score of the 13 included studies was 6.15, indicating good methodological quality overall.

**TABLE 5:** Methodological quality assessment of included studies.

Study	PEDro scale items											PEDro score
	1	2	3	4	5	6	7	8	9	10	11	
Campo et al. (2009)	0	1	0	1	0	0	0	1	1	1	1	6
Sánchez-Sixto et al. (2021)	1	1	0	1	0	0	0	1	1	1	1	7
Rosas et al. (2017)	1	1	0	1	0	0	0	1	1	1	1	7
Ramírez-Campillo et al. (2016)	1	0	0	1	0	0	0	1	1	1	1	6
Ramirez-Campillo et al. (2018)	1	1	0	1	0	0	0	1	1	1	1	7
Ozbar et al. (2014)	0	1	0	1	0	0	0	1	1	1	1	6
Ozbar (2015)	0	0	0	1	0	0	0	1	1	1	1	5
Nonnato et al. (2022)	0	1	0	1	0	0	0	1	1	1	1	6
Maciejczyk et al. (2021)	1	1	0	1	0	0	0	1	1	1	1	7
Kale (2016)	0	0	0	1	0	0	0	1	1	1	1	5
Gjinovci et al. (2017)	0	0	0	1	0	0	0	1	1	1	1	5
Fischetti et al. (2019)	1	1	0	1	0	0	0	1	1	1	1	7
Cherni et al. (2021)	1	0	0	1	0	0	0	1	1	1	1	6

Note: 1: Description of inclusion criteria; 2: Random allocation; 3: Concealed allocation; 4: Baseline similarity; 5: Blinding of participants; 6: Blinding of therapists; 7: Blinding of assessors; 8: Follow-up completeness; 9: Intention-to-treat analysis; 10: Between-group statistical results; 11: Point measures and measures of variability.

### Risk of bias

**FIGURE 2:**
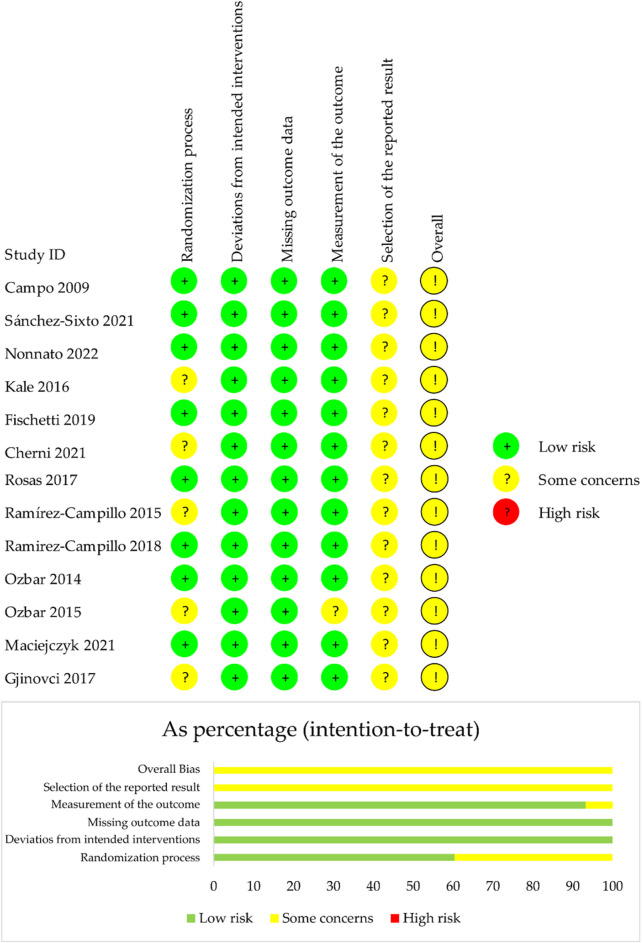
Overall risk of bias presented as percentage of each risk of bias item across all included studies.

Overall, all included studies were rated as having a moderate risk of bias. Specifically, eight studies (61.5%) reported the randomization process, while five (38.5%) did not mention the randomization process. One study (7.7%) was rated as having a moderate risk of bias in the domain of outcome measurement. The selection of reported results was unclear for all included studies and was therefore marked as “some concerns.” For detailed information, refer to Figure 2.

### Publication bias

**FIGURE 3:**
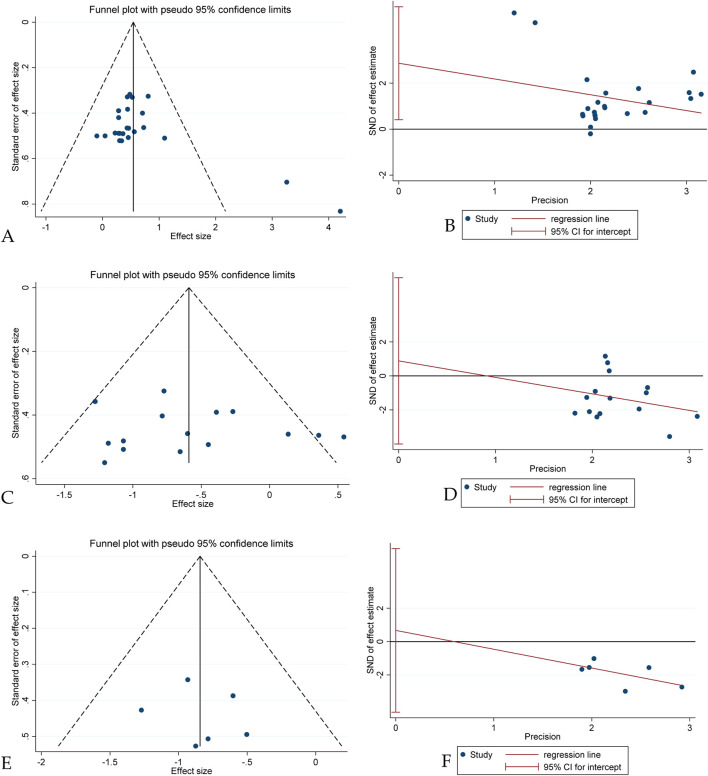
Visualization of Publication and Bias, Egger’s Test [**(A)** Funnel plot for jumping performance; **(B)** Egger’s Test for jumping performance; **(C)** Funnel plot for sprinting performance; **(D)** Egger’s Test for sprinting performance; **(E)** Funnel plot for COD performance; **(F)** Egger’s Test for COD performance].

Visual inspection of the funnel plots suggested potential publication bias in studies reporting on jumping and sprinting performance (Figure 3). Egger’s test confirmed publication bias for jumping performance (t = 2.42, p = 0.024). However, the trim-and-fill method indicated that no additional studies needed to be added, providing some support for the stability of the observed effects. No publication bias was detected for sprinting performance (t = 0.39, p = 0.703) or COD performance (t = 0.38, p = 0.723).

### Meta-analysis results

#### Jump performance

**FIGURE 4:**
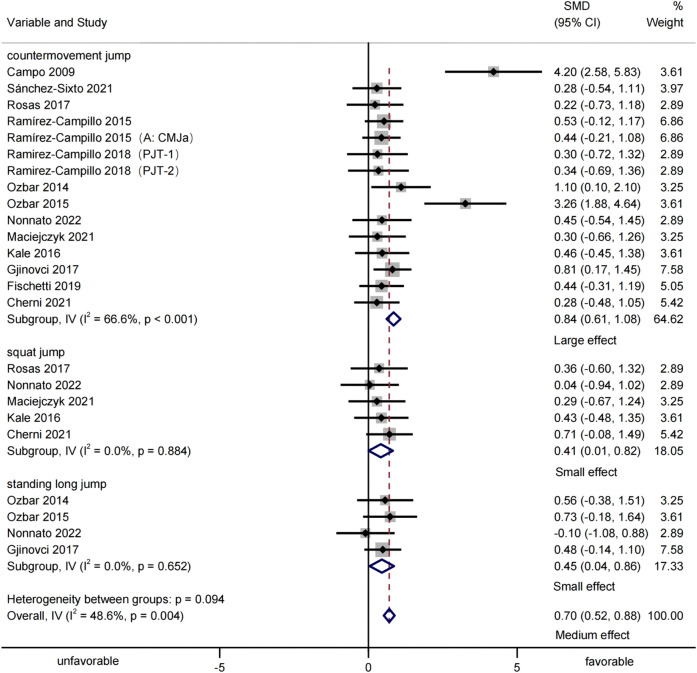
Forest-plot-based visualization of jumping-performance outcomes.

Thirteen studies were included, providing 24 groups of jumping-test data to assess the effects of plyometric training on adult female athletes’ jumping performance (Figure 4). Meta-analysis showed that plyometric training significantly improved jumping performance [SMD = 0.70, 95% CI (0.52, 0.88), p < 0.001, medium effect], with moderate heterogeneity (I^2^ = 48.6%, p = 0.004). Subgroup analysis indicated significant improvements in CMJ [SMD = 0.84, 95% CI (0.61, 1.08), p < 0.001, I^2^ = 66.6%, large effect], SJ [SMD = 0.41, 95% CI (0.01, 0.82), p = 0.046, I^2^ = 0.0%, small effect], and SLJ [SMD = 0.45, 95% CI (0.04, 0.86), p = 0.031, I^2^ = 0.0%, small effect]. For the CMJ subgroup, the stepwise exclusion method found that after excluding Campo et al. (2009), heterogeneity reduced (I^2^ = 33.6%, p = 0.106) without result reversal [SMD = 0.65, 95% CI (0.42, 0.87), p < 0.001]. Overall heterogeneity was non-significant (I^2^ = 2.9%, p = 0.422), and the overall result remained unchanged [SMD = 0.57, 95% CI (0.39, 0.75), p < 0.001]. Thus, Campo et al. (2009) was the main heterogeneity source, but the results remained robust despite heterogeneity (Table 6).

**TABLE 6:** Results of stepwise exclusion method.

Study	Elimination of within-subgroup heterogeneity	SMD within subgroups after elimination	Overall heterogeneity after elimination	Overall SMD after elimination
Campo et al. (2009)	33.6%, p = 0.106	0.65 (0.42, 0.87)	2.9%, p = 0.422	0.57 (0.39, 0.75)
Sánchez-Sixto et al. (2021)	68.6%, p < 0.001	0.88 (0.64, 1.12)	50.4%, p = 0.003	0.72 (0.53, 0.90)
Rosas et al. (2017)	68.5%, p < 0.001	0.87 (0.63, 1.11)	50.3%, p = 0.003	0.71 (0.53, 0.90)
Ramírez-Campillo et al. (2016)	69.2%, p < 0.001	0.88 (0.63, 1.13)	51.0% p = 0.003	0.71 (0.52, 0.90)
Ramírez-Campillo et al. (2016) (A: CMJa)	69.0%, p < 0.001	0.89 (0.64, 1.14)	50.9%, p = 0.003	0.72 (0.53, 0.91)
Ramirez-Campillo et al. (2018) (PJT-1)	68.8%, p < 0.001	0.87 (0.63, 1.11)	50.6%, p = 0.003	0.71 (0.53, 0.90)
Ramirez-Campillo et al. (2018) (PJT-2)	68.9%, p < 0.001	0.87 (0.63, 1.11)	50.7%, p = 0.003	0.71 (0.52, 0.90)
Ozbar et al. (2014)	68.5%, p < 0.001	0.83 (0.59, 1.07)	49.5%, p = 0.004	0.69 (0.50, 0.87)
Ozbar (2015)	48.4%, p = 0.022	0.70 (0.47, 0.94)	21.2%, p = 0.178	0.60 (0.42, 0.79)
Nonnato et al. (2022)	69.0%, p < 0.001	0.86 (0.62, 1.10)	50.8%, p = 0.003	0.71 (0.52, 0.89)
Maciejczyk et al. (2021)	68.8%, p < 0.001	0.87 (0.63, 1.11)	50.6%, p = 0.003	0.71 (0.53, 0.90)
Kale (2016)	69.1%, p < 0.001	0.87 (0.63, 1.11)	50.9%, p = 0.003	0.71 (0.52, 0.89)
Gjinovci et al. (2017)	69.1%, p < 0.001	0.85 (0.60, 1.10)	50.3%, p = 0.003	0.69 (0.50, 0.88)
Fischetti et al. (2019)	69.0%, p < 0.001	0.88 (0.63, 1.12)	50.9%, p = 0.003	0.71 (0.52, 0.90)
Cherni et al. (2021)	68.7%, p < 0.001	0.90 (0.65, 1.14)	50.5%, p = 0.003	0.72 (0.53, 0.91)

SMD, standardized mean difference; PJT-1, one session plyometric jump training per-week; PJT-2, two session plyometric jump training per-week; CMJa, countermovement jump with arms.

#### Sprint performance

**FIGURE 5:**
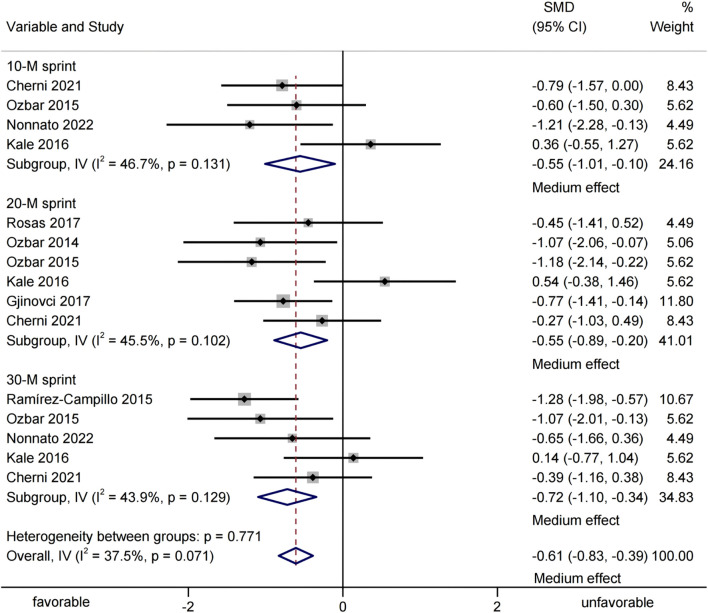
Forest-plot-based visualization of sprinting-performance outcomes.

Eight studies were included, providing 15 groups of sprint-test data to assess the effects of plyometric training on adult female athletes' sprinting performance (Figure 5). Meta-analysis showed that plyometric training significantly improved sprinting performance [SMD = −0.61, 95% CI (−0.83, −0.39), p < 0.001, medium effect], with non - significant heterogeneity (I^2^ = 37.5%, p = 0.071). Subgroup analysis indicated significant improvements in 10 - m [SMD = −0.55, 95% CI (−1.01, −0.10), p = 0.016, I^2^ = 46.7%, medium effect], 20 - m [SMD = −0.55, 95% CI (−0.89, −0.20), p = 0.002, I^2^ = 45.5%, medium effect], and 30 - m sprints [SMD = −0.72, 95% CI (−1.10, −0.34), p = 0.002, I^2^ = 43.9%, medium effect].

#### Change-of-direction performance

**FIGURE 6:**
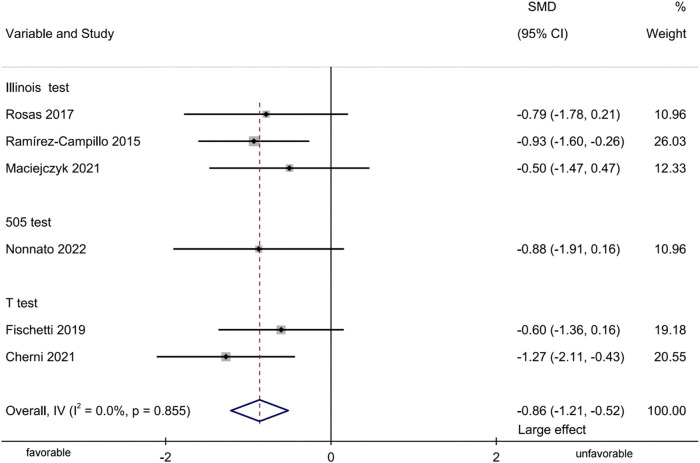
Forest-plot-based visualization of COD-performance outcomes.

Six studies were included, providing six groups of COD-test data to assess the effects of plyometric training on adult female athletes’ COD performance (Figure 6). Meta-analysis showed that plyometric training significantly improved COD performance [SMD = −0.86, 95% CI (−1.21, −0.52), p < 0.001, large effect), with no heterogeneity observed (I^2^ = 0.0%, p = 0.855). Notably, the presentation of COD performance data here reflects subgrouping by test indicators rather than formal subgroup analyses. Given the small number of studies (n = 6) contributing to this subgroup, the certainty of these findings is limited.

### Sensitivity analysis

The sensitivity analysis results indicated that for jump performance, the studies by Campo et al. (2009) (SMD = 0.50) and Ozbar (2015) (SMD = 0.50) introduced considerable deviations. However, the overall results did not exhibit bias or reversal. For sprint performance and COD performance, all results did not introduce substantial deviations, suggesting that despite the presence of heterogeneity and some concerns regarding the risk of bias, these factors did not undermine the robustness of the results. This finding indicates that the results are relatively robust. The sensitivity analysis results have been uploaded to the supplementary materials (S4-Sensitivity Analysis Results) for reference.

## Discussion

Based on the included study data, the present study further quantified, via meta-analysis, the overall effects of plyometric training on adult female athletes, and the meta-analysis results indicated that: plyometric training is a highly effective conditioning method for adult female athletes, significantly enhancing jumping (SMD = 0.70, p < 0.001, medium effect), sprinting (SMD = −0.61, p < 0.001, medium effect), and COD performance (SMD = −0.86, p < 0.001, large effect). Subgroup analyses show significant improvements in CMJ (SMD = 0.84, p < 0.001, large effect), SJ (SMD = 0.41, p = 0.046, small effect), and SLJ (SMD = 0.45, p = 0.031, small effect) for jumping, and in 10-m (SMD = −0.55, p = 0.016, medium effect), 20-m (SMD = −0.55, p = 0.002, medium effect), and 30-m (SMD = −0.72, p = 0.002, medium effect) sprints. However, due to the limited number of studies on COD performance, subgroup pooled effects were not calculated. These findings carry substantial importance as they validate plyometrics as a pivotal tool for female athletes, addressing the longstanding gap in sex-specific performance enhancement research. For training regimens, this underscores the need to integrate such protocols tailored to women’s neuromuscular profiles, ensuring optimized gains in key athletic capacities.

The efficacy of plyometric training in enhancing sport performance has been well-established, with its significant positive effects on jumping, COD, and sprinting performance supported by numerous studies (Ramirez-Campillo et al., 2020a; Chen et al., 2023a; Ramirez-Campillo et al., 2020b). While previous research has sought to generalize its applicability across different sex (Chen et al., 2025a; Stojanović et al., 2017), ages groups (Ramírez-Campillo et al., 2016; Vetrovsky et al., 2019), and training levels (Ramirez-Campillo et al., 2018; Deng et al., 2024; Huang et al., 2023; Chelly et al., 2014), much of this evidence stems from male-centric samples—such as the work by Moran et al. (2024) demonstrated its benefits in improving jumping, sprinting, and COD abilities in adult male soccer players. Despite differences in study populations, the consistent core outcomes highlight the generalizability of plyometric training across various athletic abilities. This generalizability is attributed to several neuromuscular adaptations, including enhanced SSC efficiency (i.e., improved elastic energy storage during eccentric lengthening, rapid release during concentric shortening, and faster phase transitions to amplify force output), altered activation strategies of agonist and antagonist muscles, improved muscle excitability due to SSC (Chen et al., 2023b; Stojanović et al., 2017; Markovic and Mikulic, 2010; Ullrich et al., 2018; Blazevich et al., 2003; Ullrich et al., 2018; Grosset et al., 2009; Kubo et al., 2007; Cornu et al., 1997; Fouré et al., 2009), and changes in muscle structure, such as increased muscle volume (McGarrigal et al., 2025; Edwén et al., 2014).

However, the underrepresentation of female subjects in sports science research continues to obscure the definitive effects of plyometric training on female participants (Paul et al., 2023). As early as 2019, Moran et al. (2019) highlighted the scarcity of research on plyometric training for females. Although their study confirmed its effectiveness in enhancing jumping performance in adolescent females, they also noted that unpublished and non-peer-reviewed studies dominated their collected literature. This underscores the paucity of research on females and limits the interpretability and strength of evidence in their findings. As of 2025, Chen et al. (2025a) continued to advocate for the importance of female-focused research in their study on plyometric training. Building on Moran et al. (2019), Chen et al. (2025a) further showed that plyometric training enhances jumping performance in adolescent females and improves sprinting performance. In response to the calls from Moran et al. (2019) and Chen et al. (2025a), this systematic review and meta-analysis focuses on female participants. Unlike their studies, which primarily targeted adolescent females, this review and meta-analysis specifically concentrated on adult female athletes. This work serves as a comprehensive supplement to the existing literature on plyometric training in females. Unlike previous studies, this systematic review and meta-analysis provide a holistic assessment of the effects of plyometric training on jumping, sprinting, and COD performance in adult female athletes. Our findings confirm the overall efficacy of plyometric training in enhancing these sports performance in adult female athletes, thereby further enriching the research landscape in this domain.

When discussing female subjects, it is inevitable to compare their training adaptations with those of male subjects. Although the number of studies included in this systematic review is limited, this an interesting and worthwhile topic for discussion. However, to our knowledge, only two similar studies currently exist. Thus, conclusions regarding sex differences in training adaptations between males and females, and their underlying mechanisms are extremely limited. Ramírez-Campillo et al. (2016) compared the training adaptations of male and female soccer players following plyometric training. Their results indicated that plyometric training significantly enhanced jumping, sprinting, and COD performance in both male and female participants, with no significant differences observed between the groups. The only potential difference may be that females exhibited superior potential injury-reducing effects compared to males (Ter Stege et al., 2014). In another study, Marzouki et al. (2022) compared the sex differences in the effects of plyometric training on school-aged children and adolescents. Similar to the findings of Ramírez-Campillo et al. (2016), they observed no significant differences between male and female participants in the U8 and U10 age groups. However, among the U12 population, male adolescents demonstrated significantly greater adaptations to plyometric training than their female counterparts. The normal physiological development of females may explain this phenomenon. Specifically, U12 girls may enter puberty, during which increased estrogen secretion can lead to an increase in fat tissue, thereby reducing cardiopulmonary efficiency. Fat mass represents an inert, non-contributing load that may impair biomechanical movement efficiency and potentially hinder motor proficiency (Panayiotes et al., 2012; Dumith et al., 2010; D’Hondt et al., 2009). Although no specific recommendations are available solely for females, the study by Chen et al. (2023b) provides comprehensive training suggestions. For enhancing CMJ performance, the total number of ground contacts should be limited to fewer than 900, with a recommended total training time of 500–600 min. In contrast, for improving SJ performance, the total number of ground contacts should exceed 1,400, with a total training time of 400–500 min.

In sports science, heterogeneity among studies is often inevitable (Chen et al., 2025b), as seen in the moderate heterogeneity in the CMJ subgroup for jumping performance here. Though both the overall jumping group and CMJ subgroup showed moderate heterogeneity, the leave-one-out method revealed excluding Campo et al. (2009) eliminated significant heterogeneity in both. Notably, the results remained consistent, indicating that the study by Campo et al. (2009) was the primary source of heterogeneity. Thus, even with the presence of heterogeneity in both the overall and subgroup analyses, the robustness of the meta-analysis results was maintained. Similarly, the sensitivity analysis also demonstrated that, despite the presence of heterogeneity and some concerns regarding the risk of bias, no significant deviations or reversals were observed in the overall validity of the results, thereby confirming the overall stability of our findings.

## Limitations and future direction

In this systematic review and meta-analysis, the limited number of included studies restricted our analysis to independently examining the efficacy of plyometric training on sprinting, jumping, and COD performance in adult female participants. This limitation precluded a comprehensive assessment of potential sex differences in the effects of plyometric training between male and female subjects. Consequently, the unique impact of sex on training outcomes may have been obscured. Additionally, the scarcity of studies involving healthy adult females who are not athletes meant that our statistical analysis could not include this representative and broadly applicable population. This diminishes the generalizability of our findings to the general population and highlights a gap in current research focus. Furthermore, the included studies exhibited substantial variability in intervention protocols—such as differences in training intensity, duration, frequency, and specific plyometric exercises—and critical gaps in reporting key training details (e.g., progression models, rest intervals, or exercise form standardization), which not only introduces heterogeneity but also limits our ability to link specific training components to outcomes. Another limitation is the omission of certain performance tests commonly used in plyometric research (e.g., reactive strength index tests, drop jump height at various loads) from the included studies, which restricted our ability to analyze a broader spectrum of neuromuscular adaptations. Additionally, subgroup analyses were constrained by small sample sizes in specific subgroups (e.g., sprint distances <10 m, advanced-level athletes), reducing statistical power to detect meaningful differences. Methodological differences in performance testing across studies also pose challenges: for CMJ specifically, variations in testing protocols (e.g., countermovement depth, arm swing allowance, or equipment used for force/velocity measurement) were not consistently reported, which may introduce bias in pooled outcomes. More broadly, inconsistencies in timing of assessments (e.g., post-training testing windows) further limit comparability.

Despite the emphasis on the importance of female-focused research by Moran et al. (2019) and Chen et al. (2025a), studies on female subjects in plyometric training remain underrepresented. This neglect not only limits our understanding of how females respond to and adapt to such training but may also result in developing training programs lacking specificity and scientific rigor. Therefore, this systematic review and meta-analysis reiterate the urgent need for future research to prioritize studies involving female participants. This call to action extends beyond plyometric training to other exercise modalities, where female subjects are similarly underrepresented.

## Conclusion

The results of this systematic review and meta-analysis demonstrate that plyometric training is an effective means of enhancing jumping, sprinting, and COD performance in adult female athletes. Therefore, incorporating plyometric training into training programs represents a scientifically sound and practical approach to improve sport performance in this population.

## Abbreviations

SSC, stretch-shortening cycle; CMJ, countermovement jump; CMJa, countermovement jump with arms; SJ, squat jump; SLJ, standing long jump; VJ, vertical jump; COD, change-of-direction; 10 m, 10 m sprint; 20 m, 20 m sprint; 30 m, 30 m sprint; PT, plyometric training, min, minute; PJT-1, one session plyometric jump training per-week; PJT-2, two session plyometric jump training per-week; SMD, standardized mean difference.
