# Supplementary material for: From a female perspective: plyometric training’s impact on jump, sprint, and change-of-direction performance in adult female athletes—a systematic review and meta-analysis
Source: Front Physiol. 2025 Sep 15;16:1633089. doi: 10.3389/fphys.2025.1633089 (PMC12477254; doi:10.3389/fphys.2025.1633089)
Supplement: Supplementary file 3 [file Table1.docx]

…………………………………………………………………………………………………………………....

| Database | Web of Science (all database) | |
| --- | --- | --- |
| Search date | 10 May 2025 | |
| Search period | From the inception of database to 10 May 2025 | |
| Query number | Search strategy | Documents retrieved |
| #1 | AB=("Plyometric Exercise" OR "Exercise, Plyometric" OR "Exercises, Plyometric" OR "Plyometric Exercises" OR "Plyometric Training" OR "Plyometric Trainings" OR "Training, Plyometric" OR "Trainings, Plyometric" OR "Plyometric Drill" OR "Drill, Plyometric" OR "Drills, Plyometric" OR "Plyometric Drills" OR "Stretch-Shortening Exercise" OR "Exercises, Stretch-Shortening" OR "Exercise, Stretch-Shortening" OR "Stretch Shortening Exercise" OR "Stretch-Shortening Exercises" OR "Stretch-Shortening Drill" OR "Drills, Stretch-Shortening" OR "Drill, Stretch-Shortening" OR "Stretch Shortening Drill" OR "Stretch-Shortening Drills" OR "Stretch-Shortening Cycle Exercise" OR "Cycle Exercises, Stretch-Shortening" OR "Cycle Exercise, Stretch-Shortening" OR "Exercises, Stretch-Shortening Cycle" OR "Exercise, Stretch-Shortening Cycle" OR "Stretch Shortening Cycle Exercise" OR "Stretch-Shortening Cycle Exercises") and Preprint Citation Index (exclude – database) | 1,576 |
| #2 | AB= ("Female" OR "Woman") and Preprint Citation Index (exclude – database) | 2,230,817 |
| #3 | AB= (player OR athlete) and Preprint Citation Index (exclude – database) | 820,589 |
| #4 | AB= ("Lower limb explosive strength" OR "Explosive strength" OR "explosive force" OR "Explosive power" OR "power" OR "Countermovement jump" OR "CMJ" OR "squat jump" OR "SJ" OR "standing long jump" OR "SLJ" OR "drop jump" OR "DJ" OR "sprint performance" OR "10 m" OR "20 m" OR "30 m" OR "50 m" OR "vertical jump" OR "VJ" OR "change of direction" OR "cod" OR "t-test" OR "t-drill" OR "505test" OR "Illinois agility test" OR "5-10-5 test" OR "pro agility shuttle" OR "20-yard shuttle" OR "5-10-5 shuttle") and Preprint Citation Index (exclude – database) | 11,670,322 |
| #5 | #4 AND #3 AND #2 AND #1 and Preprint Citation Index (exclude – database) | 116 |

| Database | PubMed | |
| --- | --- | --- |
| Search date | 10 May 2025 | |
| Search period | From the inception of database to 10 May 2025 | |
| Query number | Search strategy | Documents retrieved |
| #1 | "Plyometric Exercise"[Title/Abstract] OR "exercise plyometric"[Title/Abstract] OR "exercises plyometric"[Title/Abstract] OR "Plyometric Exercises"[Title/Abstract] OR "Plyometric Training"[Title/Abstract] OR "Plyometric Trainings"[Title/Abstract] OR "training plyometric"[Title/Abstract] OR "Plyometric Drill"[Title/Abstract] OR "Plyometric Drills"[Title/Abstract] OR "stretch shortening exercise"[Title/Abstract] OR "stretch shortening exercise"[Title/Abstract] OR "Stretch-Shortening Exercises"[Title/Abstract] OR "Stretch-Shortening Drills"[Title/Abstract] OR "stretch shortening cycle exercise"[Title/Abstract] OR "stretch shortening cycle exercise"[Title/Abstract] OR "Stretch-Shortening Cycle Exercises"[Title/Abstract] | 1,045 |
| #2 | "Female"[Title/Abstract] OR "Woman"[Title/Abstract] | 1,266,752 |
| #3 | "player"[Title/Abstract] OR "athlete"[Title/Abstract] | 54,854 |
| #4 | "Lower limb explosive strength"[Title/Abstract] OR "Explosive strength"[Title/Abstract] OR "explosive force"[Title/Abstract] OR "Explosive power"[Title/Abstract] OR "power"[Title/Abstract] OR "Countermovement jump"[Title/Abstract] OR "CMJ"[Title/Abstract] OR "squat jump"[Title/Abstract] OR "SJ"[Title/Abstract] OR "standing long jump"[Title/Abstract] OR "SLJ"[Title/Abstract] OR "drop jump"[Title/Abstract] OR "DJ"[Title/Abstract] OR "sprint performance"[Title/Abstract] OR "10 m"[Title/Abstract] OR "20 m"[Title/Abstract] OR "30 m"[Title/Abstract] OR "50 m"[Title/Abstract] OR "vertical jump"[Title/Abstract] OR "VJ"[Title/Abstract] OR "change of direction"[Title/Abstract] OR "cod"[Title/Abstract] OR "t-test"[Title/Abstract] OR "t-drill"[Title/Abstract] OR "505test"[Title/Abstract] OR "Illinois agility test"[Title/Abstract] OR "5-10-5 test"[Title/Abstract] OR "pro agility shuttle"[Title/Abstract] OR "20-yard shuttle"[Title/Abstract] OR "5-10-5 shuttle"[Title/Abstract] | 656,499 |
| #5 | #4 AND #3 AND #2 AND #1 | 11 |

| Database | MedLine | |
| --- | --- | --- |
| Search date | 10 May 2025 | |
| Search period | From the inception of database to 10 May 2025 | |
| Query number | Search strategy | Documents retrieved |
| #1 | Title and Abstract ("Plyometric Exercise" OR "Exercise, Plyometric" OR "Exercises, Plyometric" OR "Plyometric Exercises" OR "Plyometric Training" OR "Plyometric Trainings" OR "Training, Plyometric" OR "Trainings, Plyometric" OR "Plyometric Drill" OR "Drill, Plyometric" OR "Drills, Plyometric" OR "Plyometric Drills" OR "Stretch-Shortening Exercise" OR "Exercises, Stretch-Shortening" OR "Exercise, Stretch-Shortening" OR "Stretch Shortening Exercise" OR "Stretch-Shortening Exercises" OR "Stretch-Shortening Drill" OR "Drills, Stretch-Shortening" OR "Drill, Stretch-Shortening" OR "Stretch Shortening Drill" OR "Stretch-Shortening Drills" OR "Stretch-Shortening Cycle Exercise" OR "Cycle Exercises, Stretch-Shortening" OR "Cycle Exercise, Stretch-Shortening" OR "Exercises, Stretch-Shortening Cycle" OR "Exercise, Stretch-Shortening Cycle" OR "Stretch Shortening Cycle Exercise" OR "Stretch-Shortening Cycle Exercises") | 940 |
| #2 | Title and Abstract ("Female" OR "Woman") | 1,219,682 |
| #3 | Title and Abstract (player OR athlete) | 152,380 |
| #4 | Title and Abstract ("Lower limb explosive strength" OR "Explosive strength" OR "explosive force" OR "Explosive power" OR "power" OR "Countermovement jump" OR "CMJ" OR "squat jump" OR "SJ" OR "standing long jump" OR "SLJ" OR "drop jump" OR "DJ" OR "sprint performance" OR "10 m" OR "20 m" OR "30 m" OR "50 m" OR "vertical jump" OR "VJ" OR "change of direction" OR "cod" OR "t-test" OR "t-drill" OR "505test" OR "Illinois agility test" OR "5-10-5 test" OR "pro agility shuttle" OR "20-yard shuttle" OR "5-10-5 shuttle") | 622,238 |
| #5 | #4 AND #3 AND #2 AND #1 | 71 |

| Database | Embase | |
| --- | --- | --- |
| Search date | 10 May 2025 | |
| Search period | From the inception of database to 10 May 2025 | |
| Query number | Search strategy | Documents retrieved |
| #1 | 'plyometric exercise':ti,ab,kw OR 'exercise plyometric':ti,ab,kw OR 'exercises plyometric':ti,ab,kw OR 'plyometric exercises':ti,ab,kw OR 'plyometric training':ti,ab,kw OR 'plyometric trainings':ti,ab,kw OR 'training plyometric':ti,ab,kw OR 'plyometric drill':ti,ab,kw OR 'plyometric drills':ti,ab,kw OR 'stretch shortening exercise':ti,ab,kw OR 'stretch-shortening exercises':ti,ab,kw OR 'stretch-shortening drills':ti,ab,kw OR 'stretch shortening cycle exercise':ti,ab,kw OR 'stretch-shortening cycle exercises':ti,ab,kw | 1,064 |
| #2 | 'female':ti,ab,kw OR 'woman':ti,ab,kw | 1,931,524 |
| #3 | 'player':ti,ab,kw OR 'athlete':ti,ab,kw | 68,050 |
| #4 | 'lower limb explosive strength':ti,ab,kw OR 'explosive strength':ti,ab,kw OR 'explosive force':ti,ab,kw OR 'explosive power':ti,ab,kw OR 'power':ti,ab,kw OR 'countermovement jump':ti,ab,kw OR 'cmj':ti,ab,kw OR 'squat jump':ti,ab,kw OR 'sj':ti,ab,kw OR 'standing long jump':ti,ab,kw OR 'slj':ti,ab,kw OR 'drop jump':ti,ab,kw OR 'dj':ti,ab,kw OR 'sprint performance':ti,ab,kw OR '10 m':ti,ab,kw OR '20 m':ti,ab,kw OR '30 m':ti,ab,kw OR '50 m':ti,ab,kw OR 'vertical jump':ti,ab,kw OR 'vj':ti,ab,kw OR 'change of direction':ti,ab,kw OR 'cod':ti,ab,kw OR 't-test':ti,ab,kw OR 't-drill':ti,ab,kw OR '505test':ti,ab,kw OR 'illinois agility test':ti,ab,kw OR '5-10-5 test':ti,ab,kw OR 'pro agility shuttle':ti,ab,kw OR '20-yard shuttle':ti,ab,kw OR '5-10-5 shuttle':ti,ab,kw | 854,855 |
| #5 | #4 AND #3 AND #2 AND #1 | 10 |

| Database | Central | |
| --- | --- | --- |
| Search date | 10 May 2025 | |
| Search period | From the inception of database to 10 May 2025 | |
| Query number | Search strategy | Documents retrieved |
| #1 | "Plyometric Exercise" OR "Exercise, Plyometric" OR "Exercises, Plyometric" OR "Plyometric Exercises" OR "Plyometric Training" OR "Plyometric Trainings" OR "Training, Plyometric" OR "Trainings, Plyometric" OR "Plyometric Drill" OR "Drill, Plyometric" OR "Drills, Plyometric" OR "Plyometric Drills" OR "Stretch-Shortening Exercise" OR "Exercises, Stretch-Shortening" OR "Exercise, Stretch-Shortening" OR "Stretch Shortening Exercise" OR "Stretch-Shortening Exercises" OR "Stretch-Shortening Drill" OR "Drills, Stretch-Shortening" OR "Drill, Stretch-Shortening" OR "Stretch Shortening Drill" OR "Stretch-Shortening Drills" OR "Stretch-Shortening Cycle Exercise" OR "Cycle Exercises, Stretch-Shortening" OR "Cycle Exercise, Stretch-Shortening" OR "Exercises, Stretch-Shortening Cycle" OR "Exercise, Stretch-Shortening Cycle" OR "Stretch Shortening Cycle Exercise" OR "Stretch-Shortening Cycle Exercises" in Title Abstract Keyword - (Word variations have been searched) | 871 |
| #2 | "Female" OR "Woman" in Title Abstract Keyword - (Word variations have been searched) | 1059187 |
| #3 | player OR athlete in Title Abstract Keyword - (Word variations have been searched) | 17906 |
| #4 | "Lower limb explosive strength" OR "Explosive strength" OR "explosive force" OR "Explosive power" OR "power" OR "Countermovement jump" OR "CMJ" OR "squat jump" OR "SJ" OR "standing long jump" OR "SLJ" OR "drop jump" OR "DJ" OR "sprint performance" OR "10 m" OR "20 m" OR "30 m" OR "50 m" OR "vertical jump" OR "VJ" OR "change of direction" OR "cod" OR "t-test" OR "t-drill" OR "505test" OR "Illinois agility test" OR "5-10-5 test" OR "pro agility shuttle" OR "20-yard shuttle" OR "5-10-5 shuttle" in Title Abstract Keyword - (Word variations have been searched) | 798 |
| #5 | #4 AND #3 AND #2 AND #1 | 141 |

| Database | SPORTDiscus | |
| --- | --- | --- |
| Search date | 10 May 2025 | |
| Search period | From the inception of database to 10 May 2025 | |
| Query number | Search strategy | Documents retrieved |
| #1 | XB "Plyometric Exercise" OR "Exercise, Plyometric" OR "Exercises, Plyometric" OR "Plyometric Exercises" OR "Plyometric Training" OR "Plyometric Trainings" OR "Training, Plyometric" OR "Trainings, Plyometric" OR "Plyometric Drill" OR "Drill, Plyometric" OR "Drills, Plyometric" OR "Plyometric Drills" OR "Stretch-Shortening Exercise" OR "Exercises, Stretch-Shortening" OR "Exercise, Stretch-Shortening" OR "Stretch Shortening Exercise" OR "Stretch-Shortening Exercises" OR "Stretch-Shortening Drill" OR "Drills, Stretch-Shortening" OR "Drill, Stretch-Shortening" OR "Stretch Shortening Drill" OR "Stretch-Shortening Drills" OR "Stretch-Shortening Cycle Exercise" OR "Cycle Exercises, Stretch-Shortening" OR "Cycle Exercise, Stretch-Shortening" OR "Exercises, Stretch-Shortening Cycle" OR "Exercise, Stretch-Shortening Cycle" OR "Stretch Shortening Cycle Exercise" OR "Stretch-Shortening Cycle Exercises" | 1,631 |
| #2 | XB "Female" OR "Woman" | 72,951 |
| #3 | XB player OR athlete | 323,031 |
| #4 | XB "Lower limb explosive strength" OR "Explosive strength" OR "explosive force" OR "Explosive power" OR "power" OR "Countermovement jump" OR "CMJ" OR "squat jump" OR "SJ" OR "standing long jump" OR "SLJ" OR "drop jump" OR "DJ" OR "sprint performance" OR "10 m" OR "20 m" OR "30 m" OR "50 m" OR "vertical jump" OR "VJ" OR "change of direction" OR "cod" OR "t-test" OR "t-drill" OR "505test" OR "Illinois agility test" OR "5-10-5 test" OR "pro agility shuttle" OR "20-yard shuttle" OR "5-10-5 shuttle" | 74,723 |
| #5 | #4 AND #3 AND #2 AND #1 | 98 |
